# Research progress on the application of nanobodies in immunity and infectious skin diseases

**DOI:** 10.3389/fimmu.2025.1705553

**Published:** 2025-12-10

**Authors:** Liguo Qiu, Fengming Hu, Xiaohua Tao

**Affiliations:** 1 Dermatology Hospital of Jiangxi Province, Nanchang, Jiangxi, China; 2 Jiangxi Provincial Clinical Research Center For Skin Diseases, Nanchang, Jiangxi, China; 3 Candidate Branch of National Clinical Research Center for Skin Diseases, Nanchang, Jiangxi, China; 4 Health Commission of Jiangxi Province (JXHC) Key Laboratory of Skin Infection and Immunity, Nanchang, Jiangxi, China; 5 The Affiliated Dermatology Hospital of Nanchang University, Nanchang, Jiangxi, China

**Keywords:** nanobody, psoriasis, single-domain antibody, immune-related skin diseases, infectious skin diseases

## Abstract

In response to the limitations of traditional therapies for immune and infectious skin diseases in terms of tissue penetration, cost, and drug resistance, nanobodies derived from camelids and containing only a single heavy chain variable domain have shown significant advantages: their small molecular weight ensures excellent skin penetration ability, the extended CDR3 domain enables precise targeting of hidden epitopes, and they have excellent stability (tolerance to extreme pH, temperature, protease) and low-cost production potential. In the treatment of immune skin diseases, nanobodies effectively synergistically block key inflammatory pathways through multivalent/multispecific design, demonstrating deep therapeutic effects beyond some traditional therapies in areas such as psoriasis, atopic dermatitis, and hidradenitis suppurativa. In the field of infectious skin diseases, it effectively blocks the process of pathogen infection by efficiently neutralizing key virulence factors (such as invasion proteins, adhesion factors, toxins) of viruses, bacteria, fungi, and parasites, and has the potential to serve as a highly specific diagnostic tool. Future research and development will focus on multi-target optimization, artificial intelligence assisted design, new transdermal/long-acting delivery systems, and precision medicine strategies, promoting nanobodies as an efficient and precise solution to revolutionize the treatment of skin diseases.

## Chapter 1: Research background

The clinical treatment of immune and infectious skin diseases currently faces multiple challenges. Traditional monoclonal antibodies (mAbs) have limited tissue penetration ability due to their high molecular weight (approximately 150 kDa), making it difficult to effectively reach deep skin lesions, especially in chronic inflammatory skin diseases such as psoriasis and atopic dermatitis, where the stratum corneum barrier further hinders drug penetration (1). At the same time, the complex structure of biologics requires the production of eukaryotic expression systems, which increases treatment costs, while the immune effects mediated by Fragments crystallizable (Fc) may trigger non-specific inflammatory reactions (2). In the field of infectious skin diseases, the rapid evolution of drug-resistant strains (such as methicillin-resistant Staphylococcus aureus) and viruses (such as herpes simplex virus) has limited the efficacy of traditional antibiotics and antiviral drugs, and there is an urgent need for new targeted treatment strategies (3).

Nanobodies (Nbs), as single domain antibody fragments, exhibit unique advantages in this context. The technical principle is derived from heavy-chain antibodies (HCAbs) naturally occurring in camelids and lacking light chains, which achieve antigen binding via a single variable domain of the heavy chain of HCAbs (VHH, Variable domain of the heavy chain of heavy-chain antibodies, ~15 kDa) (4, 5). This streamlined structure endows nanobodies with excellent penetration ability, allowing them to penetrate deep into the epidermis and hair follicle units, which are target areas that traditional antibodies cannot reach (6). Structural analysis shows that the complementary determining region CDR3 length of nanobodies is significantly longer than that of traditional antibodies. The formation of finger like protrusions can accurately identify hidden epitopes (such as enzyme active centers and conserved regions of viral capsid proteins), which is particularly important when targeting virulence factors of Staphylococcus aureus or invasion proteins of herpes virus (3).

In terms of stability, the ability of nanobodies to tolerate extreme environments provides new possibilities for local administration of skin diseases (7). The experiment proves that it can maintain its activity in the pH 3.0-9.0 range, high temperature and protease environment, and is suitable for developing topical gel or spray preparations to avoid side effects of systemic administration (6). The hydrophilic amino acids in its framework region 2 (FR2) replace the hydrophobic residues of traditional antibodies, significantly improving solubility and reducing aggregation risk, which is particularly important in the local hypertonic environment of inflammatory skin diseases (8).

Multivalent design further expands the therapeutic potential of nanobodies in immune and infectious skin diseases (9). Bispecific nanobodies can simultaneously neutralize inflammatory factors and serum albumin (such as Ozoralizumab), achieving long-lasting inhibition of the inflammatory cascade reaction (3); And Tri-specific antibodies (such as Sonelokimab) can synergistically block multiple pathway targets, and synergistically inhibit the pathological process of diseases such as psoriasis (10). In the field of infection, nanobodies target pathogen surface proteins (such as fungal β- glucan synthase), disrupt biofilm formation, and enhance the efficacy of traditional drugs (7).

**Figure 1:**
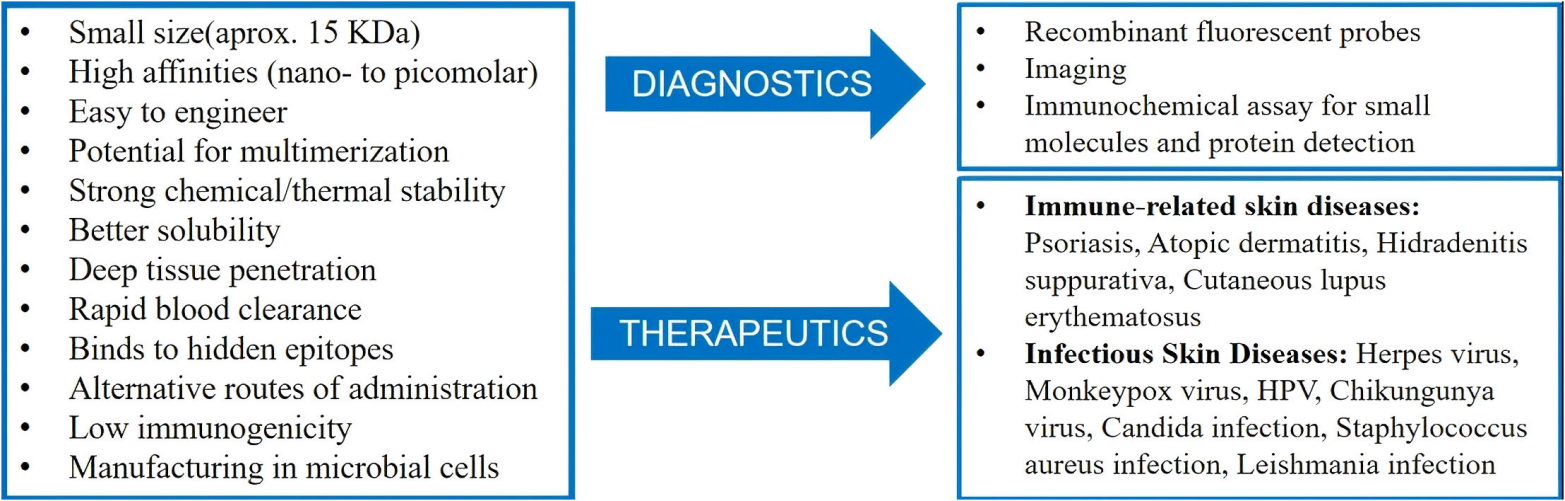
Characteristics of nanobodies and an overview of their diagnostic and therapeutic applications in immune/infectious dermatology.

Nanobodies can be efficiently expressed in prokaryotic systems, with significantly higher yields than traditional antibodies, greatly reducing production costs. With the development of machine learning assisted design (such as structure prediction and affinity optimization) and novel delivery systems (11), nanobodies are expected to reshape the treatment paradigm of immune and infectious skin diseases, providing breakthrough solutions for drug-resistant infections and chronic inflammation. **Figure 1** summarizes the functions of nanobodies and their application scenarios in immune-related infectious skin diseases.

## Chapter 2: Immune-related skin diseases

Based on the above unique advantages, nanobodies have shown great potential in the treatment and diagnosis of various immune and infectious skin diseases. Next, we will first provide a detailed review of the latest advances of nanobodies in immune-related skin diseases.

### 2.1 Psoriasis

Psoriasis, as a chronic immune-mediated inflammatory skin disease, is characterized by excessive proliferation of keratinocytes and infiltration of inflammatory cells (12). Its core pathogenesis is closely related to abnormal activation of the IL-23/Th17 axis. In recent years, nanobodies (single domain antibodies) have become an emerging force in targeted therapy for psoriasis due to their small molecular weight and high tissue permeability (13). Among them, the development process of the innovative drug Sonelokimab targeting the IL-17 pathway is particularly noteworthy (14, 15).

#### 2.1.1 Topical gel formulation: ZL-1102

From 2015 to 2017, the first anti-IL-17A nanobody gel ZL-1102 completed proof-of-concept studies (16). With a molecular weight of only 13.2 kDa, it was designed as a 1% hydrogel formulation for mild-to-moderate patients. This research constituted a randomized, double-blind, placebo-controlled Phase Ib trial (NCT06380907), divided into two parts: Part A was an open-label study where 6 patients received a single topical application to assess safety. Part B enrolled 53 patients randomly assigned to receive either 1% ZL-1102 gel or placebo twice daily for 4 weeks. Regarding safety, treatment-emergent adverse events (TEAEs) occurred at comparable rates in the ZL-1102 and placebo groups. No serious adverse events or systemic exposure (ZL-1102 not detected in plasma) were observed, with good local tolerability and no immunogenicity reactions. For efficacy, the ZL-1102 group showed a 25.3% improvement in local PASI score from baseline at Day 29, compared to 17.4% in the placebo group, though the difference was not statistically significant. Trend analysis indicated numerical improvements in erythema and scaling scores. RNA sequencing confirmed ZL-1102’s ability to penetrate the skin and downregulate pro-inflammatory gene pathways, such as those related to keratinization.

The advantage of the nanobody gel ZL-1102 lies in its topical administration avoiding systemic side effects, providing targeted therapy for mild to moderate psoriasis and filling the gap left by existing systemic IL-17 inhibitors (e.g., secukinumab) being limited to moderate-to-severe patients. Additionally, its small molecular size enhances skin permeability, and it demonstrates good short-term safety. Limitations include sub-statistical efficacy, potentially related to small sample size and short treatment duration (only 4 weeks). Biomarker changes did not fully translate into clinically significant improvements. Long-term efficacy and optimal dosage require validation in larger Phase II trials (e.g., NCT06380907 initiated in 2024) (17).

While ZL-1102 exemplifies the promise of topical nanobody delivery, the broader field of transdermal nanobody applications still faces several limitations compared to systemic or injectable formats. The stratum corneum remains a significant barrier, particularly in hyperkeratotic conditions such as chronic plaque psoriasis. Moreover, variable skin hydration and inflammation across patients may influence drug absorption and efficacy. There is also the risk of local immunogenicity or irritation with repeated application, although nanobodies generally exhibit low immunogenicity. To overcome these hurdles, future efforts should focus on advanced formulation strategies, such as enzyme-responsive hydrogels that release nanobodies specifically in inflamed skin, or microneedle-assisted delivery to enhance penetration into deeper lesions. Additionally, co-administration with permeation enhancers (e.g., ceramides or synthetic peptides) could improve skin uptake without compromising barrier function. Ultimately, the success of transdermal nanobody therapies will depend on a multidisciplinary approach combining bioengineering, dermatology, and immunology to develop patient-friendly, effective, and safe delivery systems.

#### 2.1.2 Systemic injection: sonelokimab

**Figure 2:**
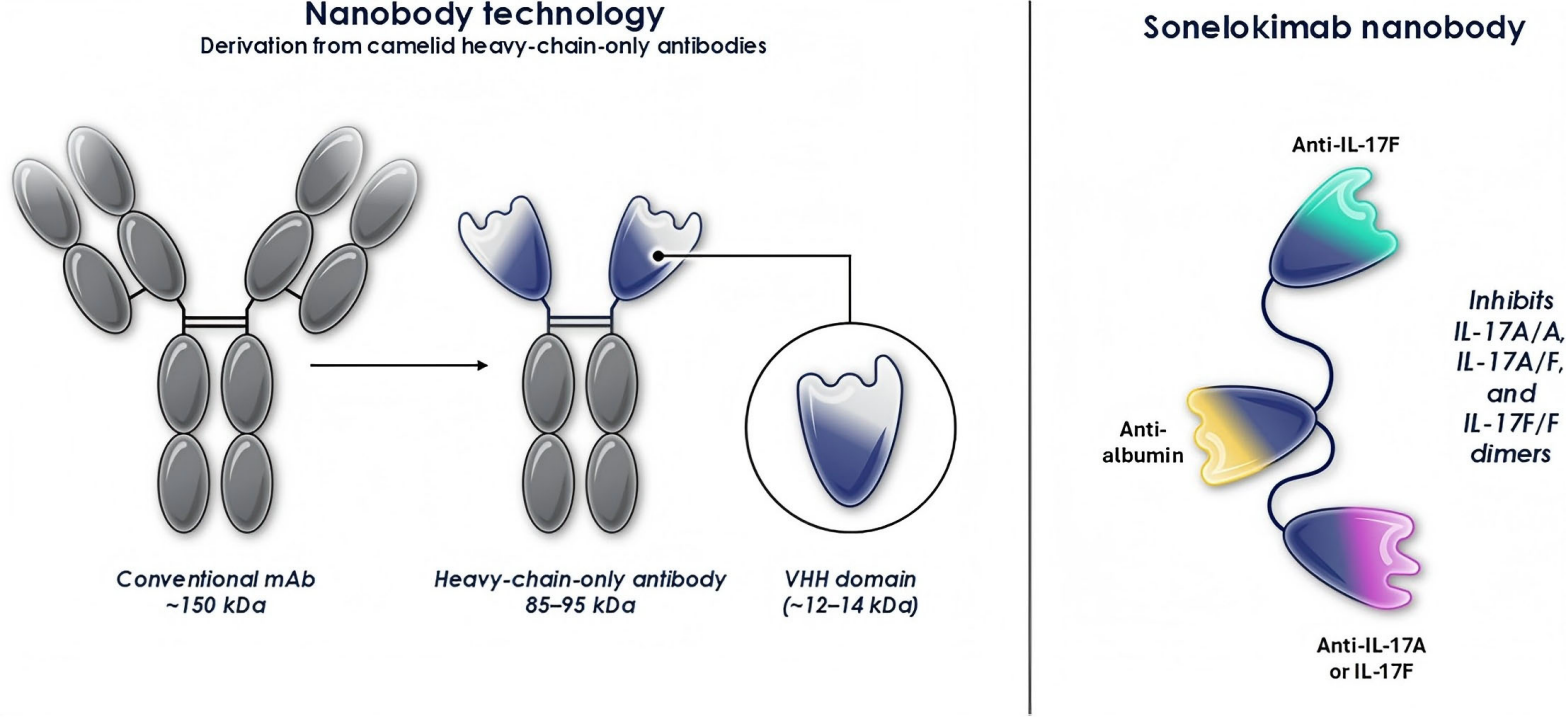
Shows a schematic diagram comparing a conventional monoclonal antibody with a heavy-chain-only camelid-derived VHH domain (i.e., a nanobody), and illustrates the trivalent nanobody Sonelokimab, which is constructed via glycine-serine linkers. Sonelokimab features an N-terminal moiety that binds to IL-17F and a C-terminal moiety that binds to both IL-17A and IL-17F.

Since 2019, the development of nanobodies has shifted towards the trivalent structure of Sonelokimab for systemic administration. Its innovative design includes: N-terminal targeting of IL-17F, central binding of serum albumin to prolong half-life, C-terminal targeting of IL-17A and IL-17F (18). **Figure 2** presents a comparison between traditional monoclonal antibodies and the heavy chain VHH portion derived solely from reindeer (i.e., nanobodies), as well as a structural schematic of the trivalent nanobody Sonelokimab.

**Figure 3:**
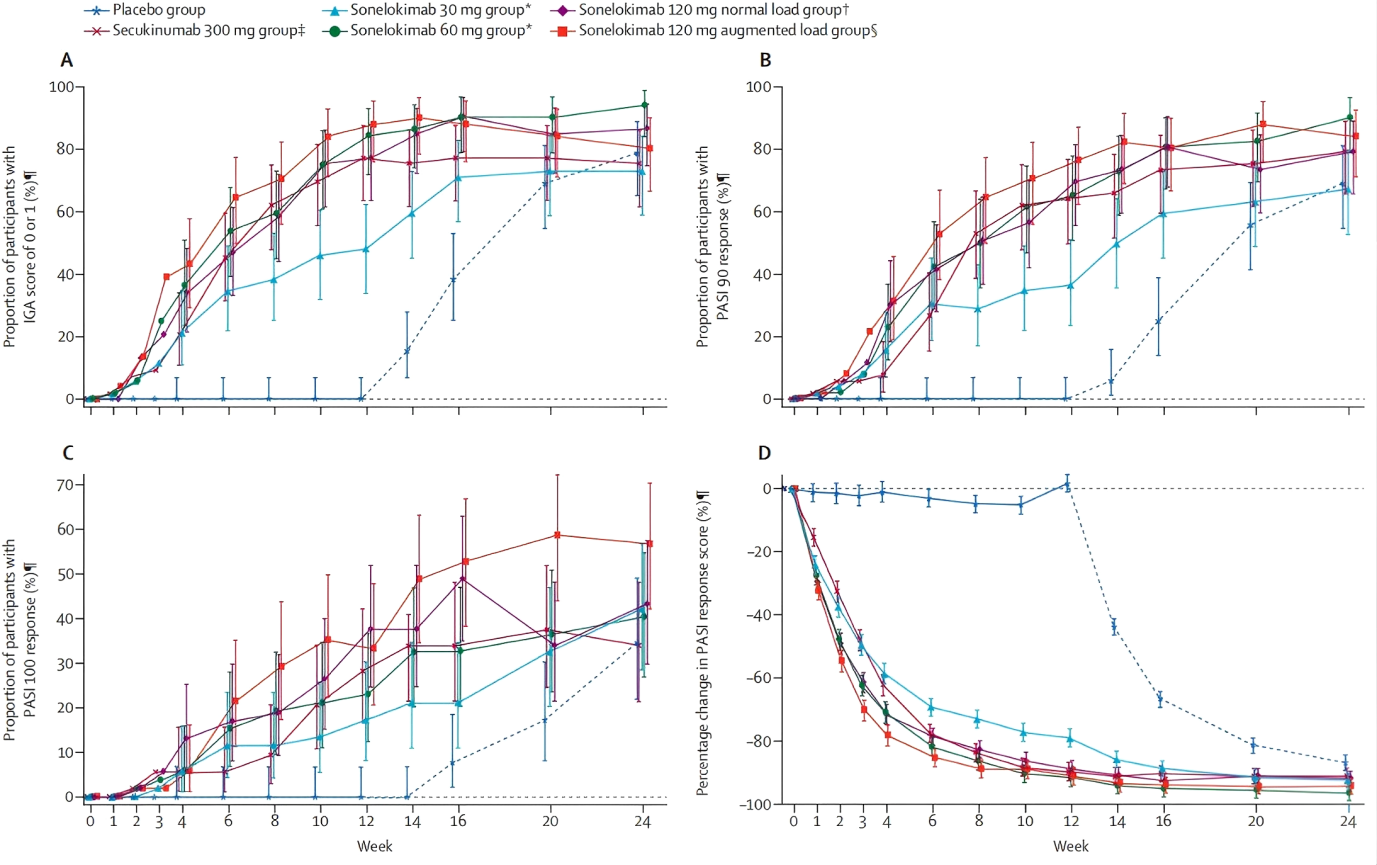
IGA and PASI response rates in the intention-to-treat population from baseline to week 24; **(A)** Proportion of participants achieving IGA 0/1; **(B)** PASI 90 response; **(C)** PASI 100 response from baseline to week 24. Missing data imputed as non-response. Error bars: 95% CIs (Clopper-Pearson method) for **(A-C)**; SEs for **(D)**. **(D)** Least-squares mean percentage change in PASI score (LOCF). Dashed lines for placebo group indicate switch to sonelokimab 120 mg every 4 weeks after week 12. IGA, Investigator’s Global Assessment; PASI, Psoriasis Area and Severity Index; PASI 90, ≥90% improvement from baseline; PASI 100, 100% improvement from baseline.

In the Phase I trial (NCT02156466), 56% of patients in the 240mg dose group achieved PASI 100, while the 2021 Phase IIb study (NCT03384745) further confirmed that after 12 weeks of treatment with the 120mg enhanced load regimen, 88.2% of patients achieved complete/almost complete clearance of skin lesions (IGA 0/1), surpassing the efficacy of traditional IL-17A monoclonal antibody Secukinumab (77.4%) (19). These results are visually verified in **Figure 3** by comparing the 24-week IGA 0/1 and PASI response rates. This advantage stems from the dual target synergistic mechanism - the expression level of IL-17F in skin lesions is 30 times higher than IL-17A, and the efficiency of IL-17A/F heterodimer activation of the keratinocyte pathway is increased by 3 times (18).

The latest clinical progress comes from the ARGO trial published in Nature Medicine in 2025, the first global Phase II study evaluating sonelokimab in active psoriatic arthritis (PsA) (20). The trial demonstrated that at 12 weeks, the sonelokimab treatment group achieved an ACR50 response rate of 46.3%–46.5%, significantly superior to the placebo group (20.0%). Key secondary endpoints, including ACR20 response rate (72.1%-78.0% vs 37.5%) and PASI 90 response rate (59.3%-76.9% vs 15.4%), both demonstrated statistically significant improvements. Efficacy deepened at 24 weeks, with the ACR50 response rate increasing to 58.1–61.0%, and 48% of patients achieving the high-threshold composite endpoint of ACR70 + PASI100. Regarding safety, sonelokimab demonstrated good tolerability. The incidence of adverse events during treatment was comparable to placebo, with the most common being nasopharyngitis (5.2%-6.1%) and upper respiratory tract infection (4.1%-6.1%). The incidence of candidiasis was low (2.1-2.4%), and no cases of inflammatory bowel disease or severe liver enzyme elevation were observed.

With the advancement of clinical applications, safety management has become a key focus (21). The Candida infection rate of systemic Sonelokimab (7.4%) is slightly higher than that of traditional drugs, which is directly related to the impaired protective effect of IL-17F in mucosal immunity (10, 22). Researchers have found that serum TSLP>2000 pg/ml (observed in critically ill patients with PASI>20) can indicate a high risk of infection, and this marker is strongly positively correlated with disease activity (r=0.86, p<0.001) (23, 24). To prolong the remission period, the latest research draws on the immune memory clearance mechanism of IL-23 inhibitors and explores the strategy of nanobodies targeting tissue resident memory T cells (22). According to data from 2022, 20% of 29 patients who achieved complete remission remained in an untreated state until 44 weeks after discontinuation of medication, but relapse patients were still able to respond efficiently after re administration (80% achieved IGA 0 again) (10).

The nanobody therapy for psoriasis demonstrates an innovative breakthrough through a cross-pathological dimensionally coordinated intervention mechanism. The anti-FGF-2 nanobody is produced optimally through the BYZYS yeast system (with a 16-fold increase in yield), and its core therapeutic value lies in targeting and inhibiting the abnormal proliferation of keratinocytes mediated by FGF-2 and the IL-1β/IL-6/IL-23 inflammatory cascade reaction (25). It simultaneously blocks both the excessive proliferation of the epidermis and the release of downstream inflammatory mediators. This strategy starts from the dimension of blocking epidermal proliferation, and through precise molecular regulation, constructs a therapeutic system covering the key pathological mechanisms of psoriasis, providing an innovative solution to overcome the limitations of traditional therapies.

Sonelokimab still presents several significant limitations in the treatment of psoriasis and psoriatic arthritis. Its safety profile exhibits characteristic risks associated with IL-17 pathway inhibition, including candidal infections and injection site reactions, necessitating continued vigilance in clinical practice. Efficacy evidence indicates limited improvement for patients with severe axial symptoms, while the 24-week observation period is insufficient for comprehensively assessing long-term safety. The suboptimal efficacy of the dosing regimen, particularly in the non-induction group, suggests a need for further optimization. These limitations indicate that the clinical utility of this drug requires comprehensive evaluation based on longer-term and broader research data.

### 2.2 Atopic dermatitis

After demonstrating strong efficacy in psoriasis, research focus has also expanded to another important inflammatory skin disease—atopic dermatitis. The application strategies of nanobodies in this area differ, placing greater emphasis on combating pathogenic factors such as Staphylococcus aureus superantigens.

Atopic dermatitis (AD) is a common chronic inflammatory skin disease characterized by impaired skin barrier function, immune dysregulation, and intense itching, significantly impacting patients’ quality of life (26, 27). Recent studies indicate that nanobodies, as novel biologics, demonstrate unique value in both the treatment of atopic dermatitis and research into its pathogenic mechanisms.

In therapeutic approaches for atopic dermatitis, nanobodies primarily exert their effects through highly efficient neutralization of specific inflammatory mediators or pathogenic antigens. For instance, nanobodies designed by Zong (28) against Staphylococcus aureus enterotoxin B (SEB)—such as Nb6 and Nb3—inhibit SEB’s superantigenic activity by structurally specific binding to its T cell receptor (TCR) or major histocompatibility complex (MHC) binding interface, thereby alleviating SEB-driven skin inflammation. As described by Zanganeh (29), anti-SEB nanobodies developed via phage display technology exhibit high affinity (affinity constants reaching 1nM) and strong specificity, while being easily expressed in large quantities in prokaryotic systems. This facilitates the development of low-cost diagnostic or therapeutic formulations.

However, nanobodies designed against atopic dermatitis-related targets have also revealed certain limitations in research. Taking Nb6 mentioned in Zong’s study as an example, despite its ability to bind two distinct epitopes of SEB simultaneously, surface plasmon resonance (SPR) data indicate a relatively high dissociation rate constant (kd = 2.2×10^-2^ s^-1^). This suggests a potentially short duration of action *in vivo*, necessitating frequent dosing to maintain efficacy. Furthermore, certain nanobodies (e.g., Nb15 and Nb18) exhibit a tendency toward oligomerization during complex formation, compromising their homogeneity and developability. For instance, heterodimer peaks observed in fluorescence size exclusion chromatography (FSEC) indicate insufficient stability. In practical applications, while nanobodies exhibit lower immunogenicity than conventional antibodies, repeated use may still induce antibody-drug reactions. Furthermore, their small-molecule nature may lead to rapid renal clearance, limiting their long-term efficacy.

### 2.3 Hidradenitis suppurativa

Hidradenitis suppurativa (HS) is a chronic, inflammatory, recurrent skin disease characterized by painful nodules, abscesses, and skin tunnel formation (30). It is closely associated with immune system dysregulation, particularly the abnormal activation of inflammatory pathways such as TNF-α and OX40L, significantly impacting patients’ quality of life. SAR442970 is a bispecific pentavalent nanobody engineered to simultaneously block both TNF-α and OX40L, two key inflammatory pathways (31). Its potential application in HS is based on the following findings: Preclinical studies indicate that combined blockade of TNF-α and OX40L more effectively controls complex immune responses than inhibiting either pathway alone. In primate models, it potently suppressed antibody production and inflammatory cell infiltration in the skin. Furthermore, transcriptomic analysis revealed that SAR442970 treatment reverses gene expression patterns highly similar to HS lesions. The significant increase in OX40-positive T cells within the affected skin of HS patients provides direct justification for targeting OX40L. Currently, this therapy has entered Phase II clinical trials to evaluate its efficacy and safety in patients with moderate-to-severe HS.

The advantage of this nanobody approach lies in its synergistic mechanism of action. By simultaneously inhibiting innate immunity (TNF-α) and adaptive immunity (OX40L-mediated T-cell and B-cell interactions), it may more comprehensively intervene in the complex pathophysiology of HS, potentially achieving greater efficacy than existing single-target therapies such as adalimumab. The serum albumin binding domain in its structure also extends the drug’s half-life *in vivo*, improving its pharmacokinetic properties.

However, disadvantages and challenges remain: as a novel therapeutic, its long-term safety and ultimate efficacy in humans require validation through large-scale clinical trials; despite low immunogenicity risk in preclinical studies, potential anti-drug antibody responses in patient populations warrant close monitoring; additionally, the development and manufacturing processes for bispecific nanobodies are relatively complex, potentially leading to higher treatment costs.

### 2.4 Cutaneous lupus erythematosus

IL-6 in Cutaneous Lupus Erythematosus (CLE) directly activates keratinocyte apoptosis, drives acute phase response, synergizes IFN-I to amplify inflammation, and promotes B/T cell-mediated autoimmune response, forming a multidimensional pathological network, which is a common pathological feature of systemic lupus erythematosus (SLE) and rheumatoid arthritis (RA) (32, 33). ALX-0061, also known as Vobarilizumab, represents a new generation of IL-6 receptor-targeted therapies, with a small molecular weight (26 kDa) and high affinity, providing new ideas for the treatment of SLE. Preclinical data supports its potential to inhibit inflammatory markers and improve joint symptoms. However, the issues of immunogenicity, efficacy, and safety have been validated through clinical studies (NCT02437890) and no further results have been obtained, indicating the possibility of research and development interruption due to undisclosed reasons. Future research needs to further focus on skin specific efficacy, combination therapy strategies, and personalized treatment guided by biomarkers to fill the gap in CLE targeted therapy. The summary of the application of Nanobodies in Immune Skin Diseases is shown in **Table 1**.

**Table 1:** Summary of nanobodies in immune skin diseases.

Disease	Nanobody name/identifier	Target(s)	Structure/type	Stage of development	Key findings/notes
Psoriasis	Sonelokimab (ALX-0761)	IL-17A, IL-17F	Trivalent (anti-IL-17A/IL-17F/Albumin)	Phase Ib (NCT03384745), Phase IIb	88.2% of patients achieved IGA 0/1 (120mg), superior to Secukinumab (77.4%). In Phase II for psoriatic arthritis (ARGO trial), ACR50 46.3–46.5% at 12 weeks.
	ZL-1102	IL-17A	Monovalent, 1% hydrogel	Phase Ib (NCT06380907)	Topical application; 25.3% improvement in local PASI vs 17.4% placebo; no systemic exposure.
	anti-FGF-2 nanobody (M-F3)	FGF-2	Monovalent	Preclinical	Produced in *Pichia pastoris* with 16x yield increase; inhibits keratinocyte proliferation.
Atopic Dermatitis	Nb6, Nb3	Staphylococcal Enterotoxin B (SEB)	Monovalent (from phage library)	Preclinical	For diagnostics and toxin neutralization. Nb6 has high dissociation rate (kd = 2.2×10^-2^ s^-1^).
Hidradenitis Suppurativa	SAR442970	TNF-α, OX40L	Bispecific Pentavalent	Phase II	Preclinical: suppresses inflammation in models; targets OX40-positive T cells.
Cutaneous Lupus Erythematosus	ALX-0061 (Vobarilizumab)	IL-6R	Monovalent	Phase I Discontinued (NCT03437890)	Development halted for undisclosed reasons.

## Chapter 3: Infectious skin diseases

The success of nanobodies in immune-mediated skin diseases demonstrates their ability to precisely modulate abnormal host immune responses. However, their application prospects go far beyond this. As we will see below, with their ability to efficiently neutralize key virulence factors of invading pathogens, nanobodies have also opened new avenues for diagnosis and treatment in the field of infectious skin diseases. The research and application of nanobodies in infectious skin diseases are summarized in **Table 2**.

**Table 2:** Summary of nanobodies in infectious skin diseases.

Pathogen	Nanobody name/identifier	Target	Key properties	Stage	Primary function
Viral infections					
HSV	Nb14-32 Fc (bispecific)	gD protein (non-overlapping epitopes)	HSV-1 IC50 = 0.51 nM; HSV-2 IC50 = 0.50 nM	Preclinical	Therapeutic: Neutralization; reduces viral load by 3-log in models.
MPXV	Bi-M1A8	Membrane protein M1R	KD=24.1 pM	Preclinical	Therapeutic: Neutralizes MPXV Clade I/II and vaccinia virus.
	Tri-M1R-01	Membrane protein M1R	Trivalent design	Preclinical	Therapeutic: 80% survival post-challenge.
	WHH-1	A35R protein	EC50 = 0.010 μg/mL	Preclinical	Diagnostic: Detection tool.
HPV	AHPVO, AHPVT	HPV16 L1 capsid protein	IC50 = 7.8 nM/6.5 nM	Preclinical	Therapeutic: Inhibits pseudovirus infection.
	2C12	HPV16 L1 capsid protein	Binds via CDR3	Preclinical	Therapeutic: Blocks viral entry.
CHIKV	Nb-A9	Non-structural protein nsP2 (aa 128-137)	Kd=174 nM	Preclinical	Therapeutic: Blocks early replication; delays cytopathy by 48h.
Fungal infections					
C. albicans	CAL1-F1	Candidalysin toxin	High affinity	Preclinical	Therapeutic: Toxin neutralization; reduces cell necrosis and inflammation.
	WHH19, WHH14	Surface adhesins (Als3/Als4)	Low nanomolar affinity	Preclinical	Diagnostic: Detection tool.
Bacterial infections					
S. aureus	CNb1	Clumping factor A (ClfA)	High affinity	Preclinical	Therapeutic: Inhibits bacterial adhesion to fibrinogen.
	VHH6	Heme receptors (IsdH, IsdB)	Dual targeting	Preclinical	Therapeutic: Inhibits iron uptake; >50% growth inhibition of MRSA.
	ICab	Resistance transporter (NorC)	Mechanism elucidated	Preclinical	Therapeutic: Inhibits efflux pump; resensitizes bacteria to antibiotics.
	Nb7, Nb1	Enterotoxin B (SEB)	Sensitivity: 0.3 ng/mL	Preclinical	Diagnostic: Detection free from Protein A interference.
	anti-CD19 nanobody	CD19 (on B-cells)	Targeting carrier	Preclinical	Therapeutic: Delivers anthrax lethal factor (LF) to induce apoptosis.
Parasitic infections					
L. tropica	NbLt05, NbLt06, NbLt14, NbLt24, NbLt36	Surface antigen (gp63)	Targets conserved epitopes	Preclinical	Diagnostic & Therapeutic: Reduces infection rate by 30-40%.

### 3.1 Viral infections

**Figure 4:**
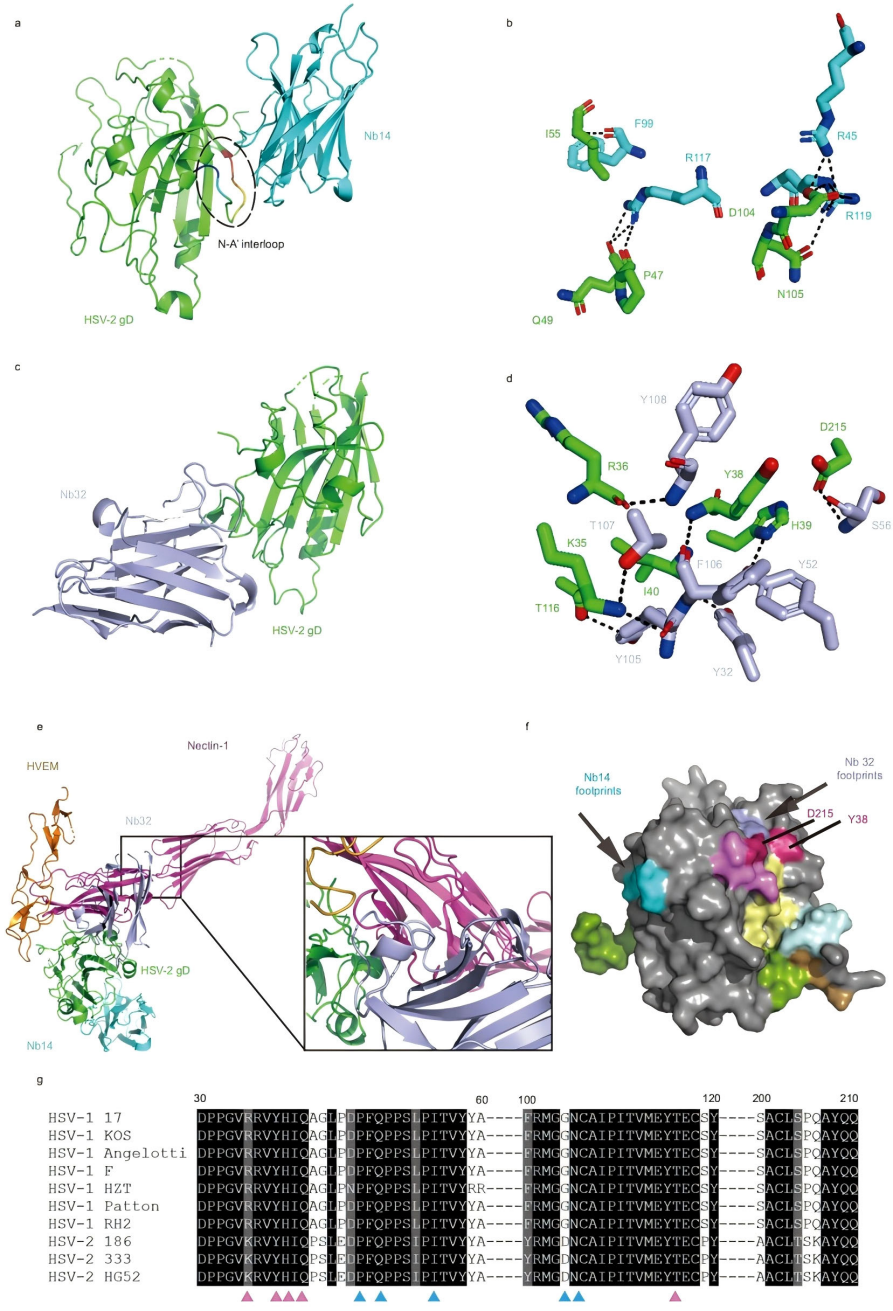
Structural basis of neutralization for Nb14 and Nb32. **(a)** Overall structure of Nb14/HSV-2 gD complex shown in cartoon. HSV-2 gD is highlighted in green and Nb14 in cyan. The N-A’ interloop is highlighted with a black dotted line. **(b)** Detailed interactions between Nb14 and HSV-2 gD. Interacting residues are shown as sticks, and dotted lines indicate hydrogen bonds and salt bridges. **(c)** Overall structure of Nb32/HSV-2 gD complex shown in cartoon. HSV-2 gD is highlighted in green and Nb32 in light blue. **(d)** Detailed interactions between Nb32 and HSV-2 gD. Interacting residues are shown as sticks, and dotted lines indicate hydrogen bonds and salt bridges. **(e)** Structural alignment of Nb14/Nb32/HSV-2 gD and Nectin-1/HSV-2 gD complexes [PDB code: 4MYW] and HVEM/HSV-1 gD complexes [PDB code: 1JMA]. Steric clash between Nb32 and Nectin-1 is highlighted. **(f)** A grayscale surface representation of the HSV-1 gD with the epitopes for Nb14 (cyan), Nb32 (light blue), E317 (pale yellow), HSV8 (limon), CH42 (pale cyan), CH43 (sand), 4A3 (violet). Epitope overlap is colored hot pink. **(g)** Conservation analysis of binding epitope in different HSV strains. The binding epitope of Nb14 is displayed in blue, and the binding epitope of Nb32 is displayed in pink.

Herpes simplex virus (HSV) can cause recurrent herpes on the skin and mucous membranes (such as oral herpes, genital herpes) and serious complications (viral keratitis, encephalitis) (34). The bispecific nanobody Nb14-32 Fc developed by Hu et al. targets the non-overlapping epitope of HSV gD: Nb14 blocks the interaction between gD gH/gL to inhibit intercellular transmission, while Nb32 competitively inhibits the binding of gD to the skin cell receptor Nectin-1. The antibody showed significant neutralizing efficacy *in vitro* (HSV-1 IC50 = 0.51 nM, HSV-2 IC50 = 0.50 nM), and reduced viral load in the brain and spinal cord by 3 logs in a mouse intracranial infection model, achieving 100% protection in a vaginal infection model (35). It is also effective against corneal infections caused by acyclovir resistant strain (KOS-V204G), significantly alleviating eye symptoms and reducing tear virus secretion by up to 2 log. The structural basis of the neutralization effect of Nb14 and Nb32 is shown in **Figure 4**.

Monkeypox virus (MPXV) infection is characterized by progressive skin rashes (macules→papules→ blisters→pustules), which can affect the facial, limb, and genital skin. Bi-M1A8 (KD = 0.0241 nM), a bivalent nanobody targeting MPXV membrane protein M1R, binds to a conserved epitope and neutralizes MPXV Clade I/II and cowpox virus. Intranasal administration reduces viral titers in mouse lungs by>1 log and improves lung tissue pathological damage (36); Tri-M1R-01, on the other hand, maintained an 80% survival rate after challenge by enhancing affinity (all control group members died) (37). In addition, A35R targeted antibody VHH-1 has high affinity (EC50 = 0.010 μg/mL), providing a new tool for the diagnosis of skin infections (38).

Human papillomavirus (HPV) is the main pathogen of skin warts (common warts, flat warts), genital lesions, and skin cancers (such as Bowen’s disease and squamous cell carcinoma) (39). Single domain antibodies targeting HPV16 L1 fusion proteins AHPVD/AHPVT (IC50 of 7.8 nM/6.5 nM, respectively) inhibit pseudo viral infection by blocking virus entry and maintain stable activity under simulated skin environmental conditions (pH 4-8, 49 °C). Molecular docking reveals that the single domain antibody 2C12 forms a hydrogen bond network with HPV16 L1 through key residues in the CDR3 region (Arg39/Thr100), enhancing its binding affinity to the viral capsid and providing a new targeted therapy strategy for HPV related skin warts and cancer (40).

Chikungunya virus (CHIKV) infection can cause systemic symptoms including papules (primarily on the trunk and limbs), often accompanied by skin manifestations such as vesicles and pruritus (41). Currently, no specific antiviral therapy exists. In a study by Deng et al., a nanobody, Nb-A9, was developed to target the CHIKV non-structural protein 2 (nsP2)—a key viral protease and helicase essential for viral replication. Nb-A9 specifically binds to an epitope within amino acids 128-137 (HYE-KMRTTN) of nsP2 with high affinity (Kd = 174 nM). Stable expression of Nb-A9 in HEK293T cells demonstrated that it blocks early viral replication, delays virus-induced cytopathic effects by 48 hours, and reduces the expression of viral E1/E2 proteins and RNA load. Consequently, it alleviates the progression of skin rashes. Owning to its low molecular weight (approximately 15 kDa), Nb-A9 presents a promising candidate for developing novel transdermal delivery systems aimed at treating CHIKV-associated skin disorders (42).

### 3.2 Fungal infections

In addition to combating viruses, nanobodies also show unique value in treating skin and mucosal infections caused by fungi (such as Candida), with their mechanism mainly focused on neutralizing toxins or targeting surface adhesins.

Candida albicans infection not only causes gynecological vulvovaginal candidiasis (VVC), but also leads to skin erythematous papules, erosions, and paronychia (43). Its core mechanism is the secretion of the toxin candidelysin, which destroys the epithelial barrier (inducing calcium influx and increased membrane permeability) and drives inflammatory responses with surface adhesins (Als3/Als4); In response to this, Valentine et al. developed therapeutic nanobodies (such as CAL1-F1) that neutralize Candida albicans lysins with high affinity, significantly reducing epithelial cell necrosis (decreased LDH release) and inflammatory cytokine (IL-8/GM-CSF) release. Delayed administration is still effective, and it has a synergistic effect with fluconazole (44).

The diagnostic antibodies developed by Giuseppe et al. (such as VHH19/VHH14) specifically target fungal surface proteins (yeast polar Als4 or hyphal Als3), achieving low nanomolar affinity binding. Although drug-resistant strains have not been distinguished, their small molecular weight (≈ 15 kDa) characteristics provide a new direction for the development of transdermal delivery systems for the treatment of skin lesions. The established VHH library lays the foundation for the development of multi-target detection kits (45).

### 3.3 Bacterial infections

Shifting from the fungal to the bacterial field, nanobodies offer novel solutions to the increasingly severe problem of antibiotic resistance (such as methicillin-resistant Staphylococcus aureus, MRSA), with mechanisms encompassing inhibition of bacterial adhesion, blocking nutrient uptake, and even reversing resistance.

Nanobodies can effectively inhibit the infection process of Staphylococcus aureus by specifically binding to key virulence factors. nanobodies targeting bacterial surface adhesion factor ClfA (Clumping factor A), such as CNb1, were obtained through yeast display technology screening (46). Their affinity significantly inhibits the binding of bacteria to host fibrinogen (Fg), thereby hindering bacterial colonization. Structural simulation shows that the complementary determining region (CDR3) of CNb1 directly occupies the binding site between ClfA and Fg (such as residues Ala254, Thr256, etc.), blocking bacterial adhesion through steric hindrance effect. Similarly, nanobodies targeting IsdH and IsdB heme receptors (such as VHH6) can simultaneously inhibit the iron uptake system of Staphylococcus aureus. Experiments have shown that this dual targeted nanobody has a growth inhibition rate of over 50% against methicillin-resistant Staphylococcus aureus (MRSA) strains (USA300, MRSA49, etc.), significantly reducing the survival rate of bacteria in iron restricted environments (47).

Nanobodies targeting bacterial resistance transporters provide a new strategy for the treatment of drug-resistant bacterial infections. The mechanism of action of nanobody ICab targeting NorC transporter protein (associated with fluoroquinolone resistance) was revealed through crystal structure analysis: the CDR3 of ICab is inserted into the extracellular vesicles of NorC, blocking substrate binding and locking the transporter protein in the “outward open” conformation, inhibiting its efflux function (48). In the MRSA infection model, ICab significantly enhances the killing effect of antibiotics on drug-resistant bacteria, providing a theoretical basis for combination therapy.

Nanobodies have shown high specificity advantages in the diagnosis of Staphylococcus aureus infection. Taking the detection of staphylococcal enterotoxin B (SEB) as an example, traditional antibodies are prone to false positives due to the binding of their Fc regions to bacterial surface protein A (SpA). While it is noted that some nanobodies can potentially bind SpA via their variable domains, the specific nanobody pair employed in this study (e.g., Nb7 as the capture antibody) was demonstrated to be free from SpA interference. A sandwich ELISA method developed using this paired nanobody system (capturing antibody Nb7 + detecting antibody phage-displayed Nb1) exhibited a sensitivity of 0.3 ng/mL and a recovery rate of 87-114% for complex samples such as milk and formula. This technology provides a rapid and reliable diagnostic tool for food poisoning secondary to skin infections (49).

Nanobodies can serve as targeted carriers to enhance intracellular delivery of antibacterial drugs. Liposomes modified with anti-CD19 nanobodies can accurately deliver anthrax lethal factor (LF) to B cells. Subsequently, LF blocks the signaling pathway by cleaving MAPK kinase (MEK1/2), inducing cell apoptosis. Experiments have shown that immunoliposomes loaded with LF (ISL-LF) have a 5-fold higher killing efficiency against Raji lymphoma cells compared to free LF, and activate the mitochondrial apoptosis pathway by upregulating the expression of pro apoptotic genes such as caspase-3/8/9 (50).

### 3.4 Parasitic infections

In response to skin infections caused by Leishmania tropica, research has found that five specific nanobodies (NbLt05, NbLt06, NbLt14, NbLt24, NbLt36) can play a dual role in diagnosis and treatment by targeting conserved antigens on the parasite surface (51). At the diagnostic level, these nanobodies can cross recognize the 63 kDa and 57 kDa antigens (mainly metalloprotease gp63) of the pre flagellar body and Amastigott two-stage, and their epitope binding characteristics (confirmed by proteinase K treatment as protein derived) avoid cross reactions caused by glycosylation differences, laying the foundation for the development of high-sensitivity diagnostic tools; In terms of therapeutic mechanism, bioinformatics simulations have shown that the complementary determining region (CDR) of NbLt36 precisely binds to the 577-604 amino acid epitope of gp63 (which is highly conserved among Leishmania species), blocking the process of parasite adhesion to macrophages mediated by gp63, reducing the infection rate of parasites pretreated with nanobodies by 30% -40%, and significantly reducing the number of Amastigots in infected cells. Experiments have shown that nanobodies do not directly kill parasites, but instead reduce intracellular parasite DNA load by interfering with host pathogen interactions. These characteristics make nanobodies a dual functional molecule with both diagnostic markers and therapeutic intervention potential. Their small molecular weight (approximately 14 kDa) facilitates penetration of the skin tissue barrier, while the conservative epitope design targeting the key virulence factor gp63 provides a new direction for developing broad-spectrum anti leishmaniasis strategies.

The summary of the application of nanobodies in infectious skin diseases is shown in **Figure 2**.

## Chapter 4: Discussion and prospect

Nanobodies, with their unique molecular properties, are demonstrating the potential to revolutionize traditional diagnosis and treatment of immune and infectious skin diseases, as well as solid tumors (52). Its outstanding organizational penetrability, high affinity binding to hidden epitopes, excellent stability, and low-cost production advantages collectively constitute its core competitiveness compared to traditional antibodies. The systematic evidence reviewed in this article indicates that nanobodies have made remarkable progress in multi-target synergistic blockade of immune diseases such as psoriasis and atopic dermatitis, as well as in neutralizing key virulence factors of viral, bacterial, fungal, and parasitic infections, marking a new era of more precise and efficient skin disease treatment.

However, translating its enormous potential into widespread clinical reality still faces many challenges. The smaller molecular weight of nanobodies not only provides good penetration, but also results in a shorter half-life *in vivo*. It is urgent to optimize pharmacokinetics through methods such as fusion of albumin binding domains or Fc engineering; Although its immunogenicity has been reduced through humanized modification, the safety of long-term use still requires continuous monitoring through large-scale clinical trials. In addition, despite the obvious advantages of local administration, how to further enhance its concentration and retention time in thick stratum corneum or deep lesions through new delivery systems (such as microneedles and intelligent hydrogels) is the key to maximize its efficacy. Multispecific design, while bringing synergistic efficacy, may also introduce unforeseen off target effects or complex immune regulatory responses, which requires us to balance efficacy and safety more finely in molecular design.

Due to their small molecular weight and exceptional tissue penetration capability, nanobodies demonstrate outstanding potential in the diagnosis and treatment of solid tumors in skin or other sites (53). For instance, Li et al. demonstrated the synergistic activation of T-cell immunity through the construction of bispecific nanobodies (54). whereas Xie et al. achieved localized sustained release and reduced systemic toxicity by integrating biomaterials such as hydrogels and liposomes (55). Furthermore, in diagnostics, leveraging the rapid pharmacokinetic properties of nanobodies (half-life of 1.5-3 hours) enables advantages such as “same-day imaging” and high-resolution SPECT/PET detection (52). Future efforts should focus on optimizing renal clearance and accelerating clinical translation through humanization.

Looking ahead to the future, the development of nanobodies will increasingly rely on the cutting-edge fusion of multiple disciplines. Artificial intelligence and machine learning will be deeply involved in its structure prediction and affinity maturation process. By using machine learning to synthesize nanobodies from scratch to prepare nanobody libraries and combining them with molecular dynamics simulations, it is expected to greatly reduce the time and economic cost of constructing camel or shark antibody libraries. The integration of multiple omics data (genome, transcriptome, proteome) will help to construct precise treatment strategies guided by biomarkers and achieve personalized medication. The development of next-generation delivery technology aims to achieve controlled release and targeted delivery of drugs, which will be the core of bridging the “last mile” of local treatment. Meanwhile, “integrated diagnosis and treatment” is another promising direction, which is to develop nanobody conjugates that combine diagnostic imaging and therapeutic functions, achieving real-time monitoring and precise intervention of diseases.

In addition, exploring the combination therapy of nanobodies with traditional small molecule drugs and immunosuppressants is expected to synergistically enhance efficacy and overcome drug resistance, especially for complex and difficult to treat infectious or autoimmune skin diseases. Ultimately, promoting the rapid translation of these innovative technologies into clinical practice and developing low-cost, high thermal stability formulations to meet the medical needs of different regions around the world will be the only way for nanobodies to truly reshape the treatment landscape of skin diseases and benefit a wide range of patients.
